# Gingivitis in Pregnancy

**Published:** 1877-06

**Authors:** 


### GINGIVITIS IN PREGNANCY.

M. Pinard, in the *Gazette des Hopiteaux*, states that, having occasion to examine a great number of pregnant women at a midwifery clinic, he finds an inflamed state of the gums to be a very common occurence among them, coming on generally about the fourth month. It usually disappears a month or two after delivery, but is often prolonged over a longer period in nursing-women. It is met with also, but less frequently, in civil practice, and although the robust are not quite exempt from it, yet it is most frequently observed in women whose general health is bad. The best application consists of a solusion of chloral in equal parts of tincture of cochlearia, which is applied every, or every other, day, by means of a pencil. It causes little pain, and gives rise to a small eschar, which disappears in a day or so.

